# *Sclerotinia sclerotiorum* Agglutinin Modulates Sclerotial Development, Pathogenicity and Response to Abiotic and Biotic Stresses in Different Manners

**DOI:** 10.3390/jof9070737

**Published:** 2023-07-10

**Authors:** Yongchun Wang, Yuping Xu, Jinfeng Wei, Jing Zhang, Mingde Wu, Guoqing Li, Long Yang

**Affiliations:** State Key Laboratory of Agricultural Microbiology and Hubei Key Laboratory of Plant Pathology, Huazhong Agricultural University, Wuhan 430070, China; yongchun@webmail.hzau.edu.cn (Y.W.); yupingxu@webmail.hzau.edu.cn (Y.X.); jinfengwei@webmail.hzau.edu.cn (J.W.); zhangjing1007@mail.hzau.edu.cn (J.Z.); mingde@mail.hzau.edu.cn (M.W.); guoqingli@mail.hzau.edu.cn (G.L.)

**Keywords:** *Sclerotinia sclerotiorum*, agglutinin, SSA, *Coniothyrium minitans*, mycoparasitism

## Abstract

*Sclerotinia sclerotiorum* is an important plant pathogenic fungus of many crops. Our previous study identified the *S. sclerotiorum* agglutinin (SSA) that can be partially degraded by the serine protease CmSp1 from the mycoparasite *Coniothyrium minitans*. However, the biological functions of SSA in the pathogenicity of *S. sclerotiorum* and in its response to infection by *C. minitans,* as well as to environmental stresses, remain unknown. In this study, SSA disruption and complementary mutants were generated for characterization of its biological functions. Both the wild-type (WT) of *S. sclerotiorum* and the mutants were compared for growth and sclerotial formation on potato dextrose agar (PDA) and autoclaved carrot slices (ACS), for pathogenicity on oilseed rape, as well as for susceptibility to chemical stresses (NaCl, KCl, CaCl_2_, sorbitol, mannitol, sucrose, sodium dodecyl sulfate, H_2_O_2_) and to the mycoparasitism of *C. minitans*. The disruption mutants (Δ*SSA*-175, Δ*SSA*-178, Δ*SSA*-225) did not differ from the WT and the complementary mutant Δ*SSA*-178C in mycelial growth. However, compared to the WT and Δ*SSA*-178C, the disruption mutants formed immature sclerotia on PDA, and produced less but larger sclerotia on ACS; they became less sensitive to the eight investigated chemical stresses, but more aggressive in infecting leaves of oilseed rape, and more susceptible to mycoparasitism by *C. minitans.* These results suggest that SSA positively regulates sclerotial development and resistance to *C. minitans* mycoparasitism, but negatively regulates pathogenicity and resistance to chemical stresses.

## 1. Introduction

*Sclerotinia sclerotiorum* (Lib.) de Bary is a cosmopolitan plant pathogenic fungus with a wide range of host plants, including oilseed rape (*Brassica napus* L.), soybean (*Glycine max* Merr.) and sunflower (*Helianthus annuus* L.), causing huge economic losses for production of these crops [1,2]. It produces sclerotia, which can survive for a long time in soil or in plant debris [3]. Under humid and low temperature conditions, the sclerotia germinate to form apothecia, where ascospores are produced and discharged into the air, and finally spread onto plant tissues to initiate infection [4,5]. As a necrotrophic pathogen, the ascospores of *S. sclerotiorum* usually rely on extracellular nutrients (e.g., senescent flower petals, plant exudates from wounds) to germinate, and consequently, the germ tubes grow to form mycelia and infection cushions (e.g., appressoria-like structures) to cause infection on plant leaves, stems, pods, fruits and seeds [6,7].

Fungi have the capacity to adapt to various environmental stresses, such as oxidative and osmotic stresses in their life cycles. The cell wall of fungi can maintain cell morphology and protect cells from damage by biotic and abiotic stresses. Fungi sense environmental stresses through the cell wall integrity (CWI) signaling system, transmit the signals to the cytoplasm, and generate responses [8]. Numerous cell surface sensor proteins, including Wsc1, Wsc2, Wsc3, Mid2, and Mtl1, have been identified; they have shown the ability to sense stresses and transmit the stress-responsive signals to the Rho1 GTPase [9,10]. The activated Rho1 binds to protein kinase C (Pkc1) and activates Pkc1, which induces downstream MAP kinase (MAPK) cascade activation. Mpk1 phosphorylates and activates the transcription factor Rlm1, which in turn regulates the expression of a variety of cell wall proteins and enzymes involved in cell wall biogenesis [11,12].

Lectins are highly specific, non-catalytically active proteins that can bind reversibly to monosaccharides or oligosaccharides, and they widely exist in plants, animals, fungi, and bacteria [13,14]. Lectins bind to the cell wall mannans of adjacent cells via hydrogen bonds to form cell aggregates that are broken in the presence of specific sugars [15]. They have the ability to mediate processes such as recognition of cellular signaling, differentiation, host–pathogen interactions, and tissue transfer [16]. In yeast cells, lectins are mainly located on the cell surface. Yeast lectins are synthesized early in growth and transported to the cell wall, where they are in a non-functional state and are activated at the onset of flocculation [17]. *Kluyverornyces bulgaricus* can secrete lectins into the culture medium, and the cells flocculate during growth; flocculation can be reversed by adding galactose [18]. Fungal lectins can be used as storage proteins and are involved in the morphogenesis and development of fungi. Lectins of about 17 kDa were isolated from *Botrytis cinerea*, *S. sclerotiorum*, *S. minor* and *S. trifoliorum* via affinity chromatography. The lectins from three *S. sclerotiorum* isolates were identical upon double immunodiffusion and ELISA assays, whereas the lectins of *S. trifoliorum* and *S. minor* are closely related to those of *S. sclerotiorum*. Lectins are present at very low levels in the mycelium and accumulated to ~20% of the total proteins in mature sclerotia [19]. *S. sclerotiorum* agglutinin (SSA) contains predominantly β-sheet structures, and exhibits specificity towards GalNAc. This newly discovered lectin family is structurally unrelated to any other fungal lectin families, and it is likely to be present only in ascomycetes [20]. SSA has 153 amino acids without a signal peptide. Molecular modeling of SSA showed that SSA can fold to form a β-trefoil domain that may be structurally related to the ricin-B family [21]. SSA is a homodimeric protein consisting of two identical subunits that are specific primarily towards Gal/GalNA. The difference with other lectins is that SSA contains a single carbohydrate-binding site at the site α [22]. These results suggest that SSA belongs to a new lectin subfamily with specific sequences and carbohydrate-binding properties.

Many agglutinins have strong insecticidal properties and can be used for insect control. Previous studies have shown that SSA is highly toxic to insects through inhibition of α-amylase activity [23,24,25]. However, whether or not SSA has other functions (such as sclerotial development and resistance to ROS and other chemical stresses, as well as to other fungi) remains unknown.

*Coniothyrium minitans* Campbell is a mycoparasitic fungus of *S. sclerotiorum*, *S. minor*, and *S. trifoliorum* [26]. It is well recognized that the cell wall of the ascomycetous fungi, including *S. sclerotiorum*, is composed of chitin, glucans, protein, and mannans [27,28]. *C. minitans* secretes cell wall-degrading enzymes such as β-1,3-glucanase, chitinase, and proteases [29,30,31]. A serine protease CmSp1 was identified in our previous study [32]. Purified CmSp1 was found to be capable of degrading the *S. sclerotiorum* agglutinin protein SSA (GenBank Acc. No. ABE97202.1), implying that SSA may play a certain role in the interaction between *S. sclerotiorum* and *C. minitans*. This study was carried out to determine the biological functions of SSA in the development and pathogenicity of *S. sclerotiorum*, and in its response to mycoparasitic infection with *C. minitans,* as well as to ambient chemical stresses.

## 2. Materials and Methods

### 2.1. Fungal Strains and Cultural Media

Four fungal strains were used in this study, including *S. sclerotiorum* wild-type (WT) strain 1980, *C. minitans* WT strain Chy-1 [33], the disruption mutant Δ*CmSp1*, and the complementary mutant Δ*CmSp1*C [34]. The cultural media used in this study included potato dextrose agar (PDA) made of fresh potato, and autoclaved carrot slices (ASC) made of fresh carrot tubers (100 g in each 250-mL glass flask). PDA was used to incubate *C. minitans* and *S. sclerotiorum*, and ASC was used to incubate *S. sclerotiorum* alone for the production of sclerotia.

### 2.2. Sequence Analysis of SSA

The amino acid residues (aa) encoded by *SSA* (*sscle_01g001830*) were inferred using the ORF Finder program in GenBank (https://www.ncbi.nlm.nih.gov/ (GenBank Acc. No. APA05413.1)) with the standard codon usage. The resulting polypeptide was compared with the agglutinin SSA (GenBank Acc. No. ABE97202.1) from *S. sclerotiorum* S1954 [21].

### 2.3. Extraction of DNA/RNA and cDNA Synthesis

The mycelium grown for 2 d on cellophane film overlays on PDA was collected. Genomic DNA (gDNA) was extracted from the mycelial samples of each fungal strain or mutant using the CTAB method [35]. The total RNA was extracted also from the mycelia of each strain or mutant using Trizol^®^ reagents (Invitrogen, Carlsbad, CA, USA). The RNA was reverse-transcripted into cDNA with the reagents in the PrimeScript RT Reagent Kit with a gDNA Eraser (TaKaRa, Dalian, China), using the protocol recommended by the manufacturer; the resulting cDNA was used for detecting the expression of *SSA* with the primer pair *SSA*f/*SSA*r (Table S1).

### 2.4. Disruption of SSA

For the disruption of *SSA*, the full length of *SSA* and its flanking sequences was downloaded from NCBI (https://www.ncbi.nlm.nih.gov/ (GenBank Acc. No. CP017814.1). The upstream and downstream DNA sequences of that gene were PCR-amplified using the primer pairs *SSA*-HyF/R and *SSA*-ygF/R, respectively, with the gDNA of WT of *S. sclerotiorum* as the template (Table S1). The resulting DNA sequences were separately ligated to the hygromycin gene vector pUCH18 [36], the inserted DNA fragments were PCR-amplified using the primers *SSA*-HyF/Hy-R and gR-F/*SSA*-ygR, using the recombinant plasmid as the template (Table S1), and the resulting DNA amplicons were sequenced for validation of insertion accuracy. Then, the amplified fragments were transformed into the protoplasts of WT of *S. sclerotiorum* using polyethylene glycol (PEG) to replace the *SSA* gene with the hygromycin gene (*Hyg*) [33]. The protoplasts were plated on the protoplast regeneration medium TB3 [37], and the emerging fungal colonies were individually picked out and incubated on PDA amended with hygromycin B (50 μg/mL) for PCR identification. Disruption of *SSA* in six mutants (Δ*SSA*-138, Δ*SSA*-175, Δ*SSA*-178, Δ*SSA*-179, Δ*SSA*-181, Δ*SSA*-225) was confirmed using Southern blotting. The gDNA from these mutants as well as the WT was digested with *Bgl* II; the DNA fragments were separated via agarose gel electrophoresis, transferred to a piece of nylon membrane film, and detected using a Biotin-labeled P1 probe with the procedure recommended by the manufacturer (GE Healthcare, Amersham Biosciences, Buckinghamshire, UK).

### 2.5. Complementation of SSA

For in situ complementation of the *SSA*-deletion mutant Δ*SSA*-178, the *SSA* upstream and downstream fragments in *S. sclerotiorum* WT were PCR-amplified using primers *SSA*-UpF/R and *SSA*-DownF/R (Table S1), respectively, and separately ligated to the neomycin vector pGNW containing the neomycin resistance gene (Supplementary Figure S1). The inserted DNA fragments were PCR-amplified using primers *SSA*-UpF/NeoR and NeoF/*SSA*-DownR (Table S1), using the recombinant plasmid DNA as the template; the resulting amplicons were then sequenced for validation of sequence accuracy. They were used to transform the protoplasts of Δ*SSA*-178 with the aid of PEG, the resulting protoplasts were plated on TB3 media for regeneration at 20 °C, and the fungal colonies were individually picked out and transferred to PDA amended with neomycin (50 μg/mL). They were identified via PCR, and expression of *SSA* was detected via RT-PCR using the specific primer pairs listed in Table S1.

### 2.6. Determination of Mycelial Growth Rates and Sclerotial Formation

The WT, the *SSA* disruption mutants (Δ*SSA*-175, Δ*SSA*-178, Δ*SSA*-225), and the complementary mutant Δ*SSA*-178C of *S. sclerotiorum* were separately inoculated on PDA in Petri dishes (9 cm in diameter) and on autoclaved carrot slices in 250-mL flasks. There were five dishes and five flasks for the WT, each mutant, and the complementary mutant. The PDA cultures were incubated at 20 °C for 24 and 48 h for observation of the colony diameter in each dish, and for 10 d for observation of sclerotial formation on each culture. The colony diameter data were used to calculate radial growth rates, expressed as mm per day (mm/d). The flask cultures were incubated at 20 °C for 20 days and the sclerotia in each flask were harvested by washing in water before being counted and weighed after air drying. For observation of sclerotial structure, sclerotinia obtained from ASC cultures were fixed and sliced, processed using the procedures described by Zhou and colleagues (2022) [37], and observed under a light microscope at 200× magnification.

### 2.7. Assay for Response to Chemical Stresses

The WT and the mutants (Δ*SSA*-175, Δ*SSA*-178, Δ*SSA*-225, Δ*SSA*-178C) of *S. sclerotiorum* were separately inoculated on PDA alone (control) and on PDA amended with NaCl (0.5 mol/L), KCl (0.5 mol/L), CaCl_2_ (0.5 mol/L), sorbitol (1 mol/L), mannitol (1 mol/L), sucrose (1 mol/L), sodium dodecyl sulfate (SDS, 0.1 mg/mL), or H_2_O_2_ (3, 5, 10 mmol/L), with five dishes (replicates) for the WT or each mutant in each medium. The cultures were incubated at 20 °C in dark for 24 and 48 h, the diameter of the colony in each dish was measured, and the data of the two measurements for the WT or each mutant in each medium was used to calculate the mycelial growth rate (*GR*). The reduced growth rate (*RGR*) was calculated as follows: *RGR* (%) = 1 − *GRT*/*GRC* × 100, where *GRT* and *GRC* represent mycelial growth rates of the WT/mutants in the presence and absence of an investigated chemical, respectively.

### 2.8. Pathogenicity Test

Seeds of *Brassica napus* ‘Zhongshuang No. 9’ were sown in plastic pots filled with the potting mix (Zhengjiang Peilei Organic Manure Manufacturing Co., Ltd., Zhengjiang, China). The trays were maintained in a growth room (20 °C, 16 h light/8 h dark) for 10 d and watered as required. The seedlings in the pots were thinned to leave one in each pot, and were further incubated for 50 d. Young, fully expanded leaves were detached from the plants and placed in five rows on moisturized paper towels in a plastic tray (52 × 33 × 7 cm, length × width × height), with five leaves in each row. Mycelial agar plugs (5 mm in diameter) were removed from 1-day-old PDA cultures of the WT or each mutant and individually inoculated on the leaves, in a row, with mycelia on the agar plugs facing the leaves, one agar plug per leaf. The tray was covered individually with transparent plastic films and placed in the growth chamber (20 °C) for two days. The diameter of the leaf lesion around each agar plug was measured. The test was repeated four times.

### 2.9. Dual Culturing

Dual cultures were performed to determine the efficacy of the *C. minitans* WT (Chy-1), the disruption mutant Δ*CmSp1,* and the complementary mutant Δ*CmSp1*C of *C. minitans* in the mycoparasitic colonization of the colonies of WT, Δ*SSA*-178, and Δ*SSA*-178C, using the procedures described by Zeng and colleagues [33]. Briefly, the dual cultures were established via inoculation of *C. minitans* first in Petri dishes (9 cm in diameter) each containing 20 mL PDA amended with bromophenol blue (0.001%, *w*/*v*), 1 cm from the rim of the dishes. The cultures were incubated at 20 °C for 4 d; then, *S. sclerotiorum* was inoculated in these *C. minitans* cultures at a 7 cm distance from the inoculation point of *C. minitans*. There were seven to eight cultures (replicates) for each combination of *C. minitans* and *S. sclerotiorum* strains or mutants. The dual cultures were further incubated at 20 °C for 12 d. Areas colonized by *S. sclerotiorum* (yellow color) and *C. minitans* (blue color) in each dual culture were observed, and the size of the blue-colored area was recorded to indicate the mycoparasitic efficacy of *C. minitans* against *S. sclerotiorum.*

### 2.10. Data Analysis

A univariate procedure in SAS 8.1 software (SAS Institute, Cary, NC, USA) was used to analyze the data on sclerotial number and weight per flask in autoclaved carrot slices, and the data on relative growth rates on PDA alone or PDA plus the stress chemicals, as well as the size values of the *C. minitans*-colonized areas in the dual cultures. The means of each parameter for WT and each mutant in single cultures, as well as for Chy-1 + WT and each of the other combinations (Chy-1 + Δ*SSA*-178, Chy-1 + Δ*SSA*-178C, Δ*CmSp1* + WT, Δ*CmSp1* + Δ*SSA*-178), were compared using Student’s *t* test at *α* = 0.05 or 0.01.

## 3. Results

### 3.1. Identity of SSA

**Figure 1:**
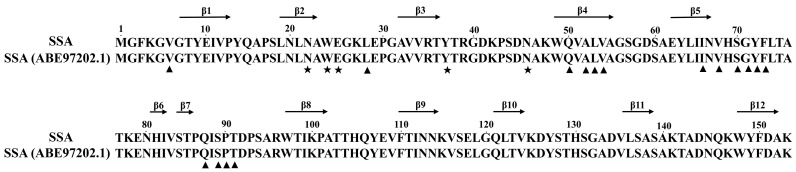
Alignment of putative amino acids of encoded by *sscle_01g001830.* SSA in *S. sclerotiorum* 1980 with the *S. sclerotiorum* agglutinin SSA (ABE97202.1). ★ and ▲ represent amino acids for the carbohydrate-binding and dimer assembly, respectively. Arrows indicate β-strands (β1 to β12).

The *SSA* gene in *S. sclerotiorum* 1980 (*sscle_01g001830*) has an open reading frame (ORF) that is 652 bp long, with three introns and four exons; it encodes a protein with 153 aa, containing the RichB_lectin_2 domain from aa 45 to aa 135 (91 aa long). It was 100% identical to the SSA in *S. sclerotiorum* S1954 (GenBank Acc. No. ABE97202.1) (Figure 1), suggesting that the protein encoded by *sscle_01g001830* is the agglutinin SSA (ABE97202.1), and *sscle_01g001830* was herein designated as *SSA*.

### 3.2. Disruption and Complementation of SSA

**Figure 2:**
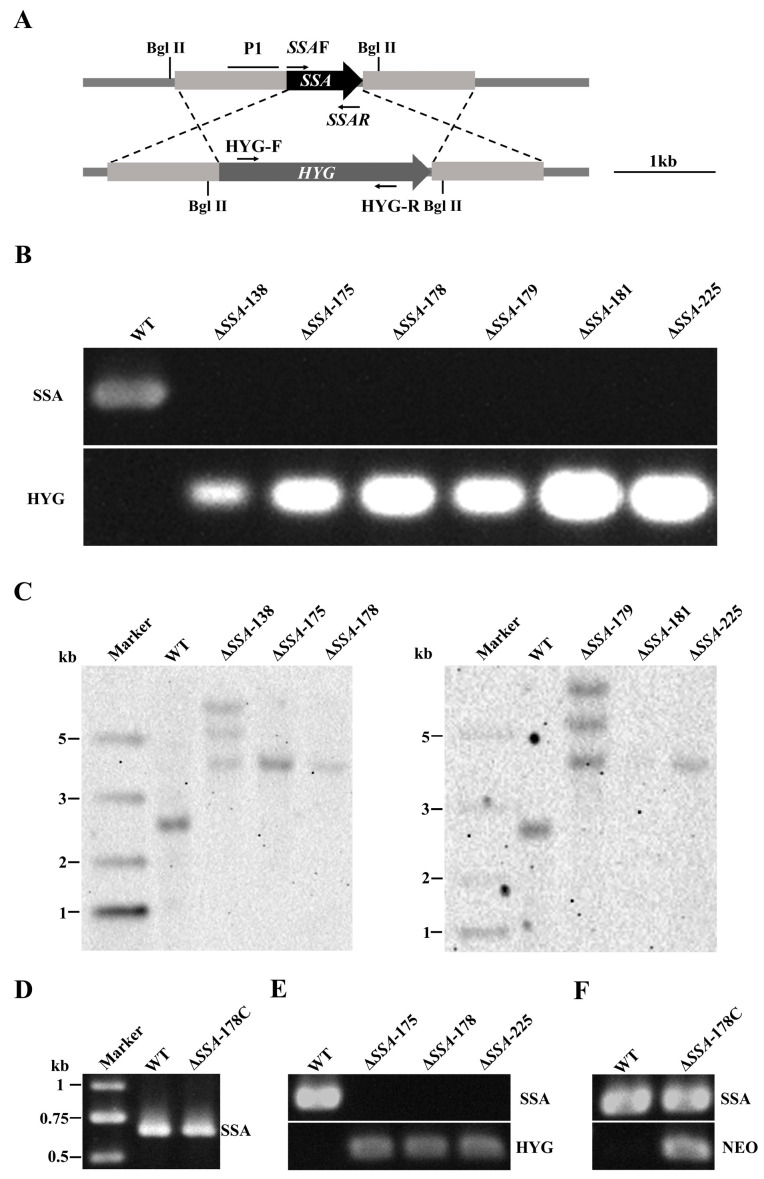
Confirmation of disruption and complementation of *SSA*. (**A**) Schematic diagram showing the strategy to disrupt *SSA*. P1, probe for Southern blotting; *Bgl* II, the restrictive digestion site; *HYG*, hygromycin resistance gene; *SSA*F/*SSA*R, the primers’ detection of *SSA*; HYG-F/HYG-R, the primers’ detection of the *HYG*. (**B**) Agarose gel electrophoregram showing PCR detection of *SSA* and the hygromycin resistance gene (*HYG*) in the WT and the disruption mutants. (**C**) Southern blotting with the probe P1l; note the different DNA bands for the WT and the disruption mutant. (**D**) Agarose gel electrophoregram showing PCR detection of *SSA* in the WT and the complementary mutant. (**E**) RT-PCR detection of the expression of *SSA* and *HYG* in the WT and the disruption mutants. (**F**) RT-PCR detection of expression of *SSA* and *NEO* in the WT and the complementary mutant.

The *SSA* gene in WT was replaced by the hygromycin gene (*Hyg*) (Figure 2A), and a total of 700 transformants showing the trait of hygromycin resistance were obtained. They were identified via PCR detection of the hygromycin resistance gene (*Hyg*) using the primer pair HYG-F/HYG-R (Table S1, Figure 2B), as well as the up and downstream regions of *SSA* using the primer pairs *SSA*-UpF/*SSA*-UpR and *SSA*-DownF/*SSA*-DownR, respectively. Six mutants (Δ*SSA*-138, Δ*SSA*-175, Δ*SSA*-178, Δ*SSA*-179, Δ*SSA*-181, Δ*SSA*-225) were finally obtained. They were further verified via PCR detection of the *SSA* ORF using the primer pair *SSA*F/*SSA*R (Table S1). The result showed that only the WT was detected to have the *SSA* ORF, whereas the six mutants did not show any positive detection of the *SSA* ORF (Figure 2B).

Southern blotting with the probe P1 (Figure 2A) indicated that the WT produced a single hybridization band of ~2.5 kb in size, whereas four of the six mutants (Δ*SSA*-175, Δ*SSA*-178, Δ*SSA*-181, Δ*SSA*-225) produced a single hybridization band of ~3.9 kb in size. As expected, Δ*SSA*-175, Δ*SSA*-178, and Δ*SSA*-225 showed a dense band, Δ*SSA*-181 showed a very faint band, and the remaining two mutants (Δ*SSA*-138, Δ*SSA*-179) produced three hybridization bands that were ~3.9, 5.1, and 6.1 kb in size (Figure 2C).

The disruption mutant Δ*SSA*-178 was transformed with the full-length ORF of *SSA* to complement *SSA* deficiency in that mutant. A mutant (Δ*SSA*-178C) showing neomycin resistance was obtained, and *SSA* was positively detected via PCR in Δ*SSA*-178C (Figure 2D). Expression of *SSA* was detected using RT-PCR in WT as well as in Δ*SSA*-175, Δ*SSA*-178, Δ*SSA*-225, and Δ*SSA*-178C. The result showed that while WT and Δ*SSA*-178C had an expression of *SSA*, the remaining three disruption mutants (Δ*SSA*-175, Δ*SSA*-178, Δ*SSA*-225) had no detectable expression of *SSA* (Figure 2E,F).

### 3.3. Effects of SSA Disruption on the Mycelial Growth Rate and Sclerotial Formation of S. sclerotiorum

**Figure 3:**
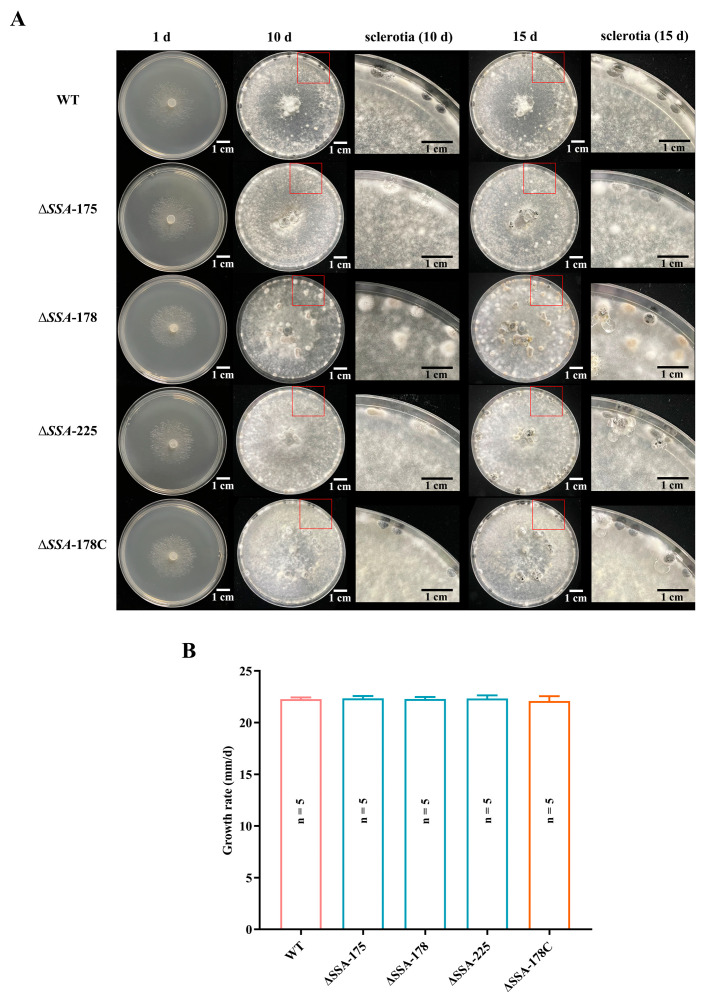
Mycelial growth of the WT as well as the disruption and complementary mutants on PDA. (**A**) Colony morphology (growth for 1 d, 10 d and 15 d at 20 °C); note the immature sclerotia formed by the disruption mutants. Red rectangles in the left panels are enlarged in the right panels. (**B**) Histogram showing mycelial growth rates; note there is no significant difference (*p* > 0.05) between the WT.

WT and the mutants (Δ*SSA*-175, Δ*SSA*-178, Δ*SSA*-225, Δ*SSA*-178C) grew rapidly on PDA at 20 °C, with the average radial mycelial growth rates ranging from 22.1 to 22.4 mm/d (Figure 3A,B), and no significant differences were detected between WT and the mutants (*p* > 0.05). After incubation for 10 d, WT and Δ*SSA*-178C formed black mature sclerotia on the colonies and at the rim of the Petri dishes (Figure 3A). However, the disruption mutants Δ*SSA*-175, Δ*SSA*-178, and Δ*SSA*-225 formed immature sclerotia with water drops on the sclerotial surface or sclerotial primordia in the colony center (Figure 3B). After incubation for 15 d, some of sclerotia in the cultures of the three disruption mutants became black, indicating that maturation of the sclerotia in the cultures of the mutants was delayed, compared to those in the cultures of WT and the complementary mutant (Figure 3B).

**Figure 4:**
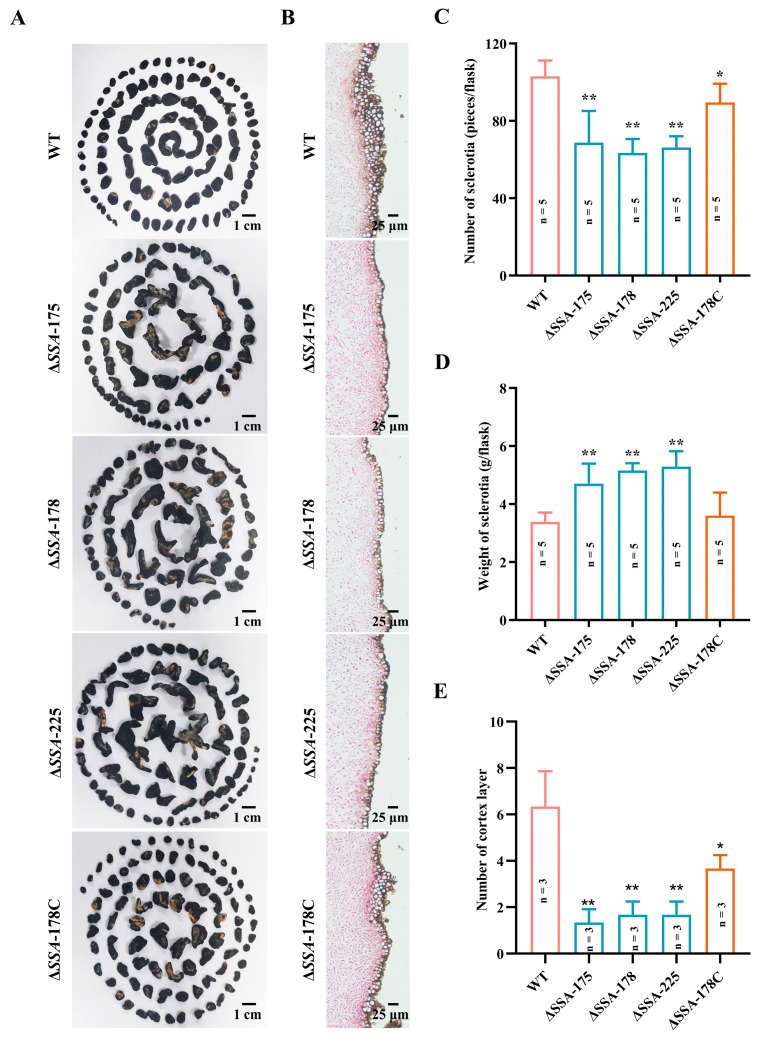
Sclerotial formation and structure of the WT as well as the disruption and the complementary mutants cultured on autoclaved carrot slices (20 °C, 20 d). (**A**) Five lots of sclerotia from five fungal cultures in 250-mL flasks; (**B**) Light microscopic graphs showing the sclerotial structure; (**C**,**D**) Two histograms showing the number of sclerotia per flask and weight of sclerotia per flask, respectively; (**E**) A histogram showing the number of layers of the sclerotinous outer cortex. * and ** represent significant differences between the WT and the investigated mutant at *p* < 0.05 and *p* < 0.01, respectively, according to Student’s *t* test.

On autoclaved carrot slices (20 °C, 20 d), the WT and the complementary mutant Δ*SSA*-178C formed 103 and 89 sclerotia per flask, respectively, with the average sclerotial weight at 3.4 and 3.6 g per flask, respectively (Figure 4A,C,D). The disruption mutants Δ*SSA*-175, Δ*SSA*-178, and Δ*SSA*-225 formed significantly (*p* < 0.01) fewer but larger sclerotia, with the average yield ranging from 60 to 68 sclerotia per flask, and the average sclerotial weight ranging from 4.7 to 5.3 g per flask (Figure 4A,C,D). Interestingly, the cortex layer (outside) of the sclerotia formed by the WT and the complementary mutant was significantly thicker than that of the disruption mutants (Δ*SSA*-175, Δ*SSA*-178 and Δ*SSA*-225) (Figure 4B,E). These results suggest that SSA plays an important role in the sclerotial development of *S. sclerotiorum*.

### 3.4. Effect of SSA Disruption on the Response of S. sclerotiorum to Chemical Stresses

**Figure 5:**
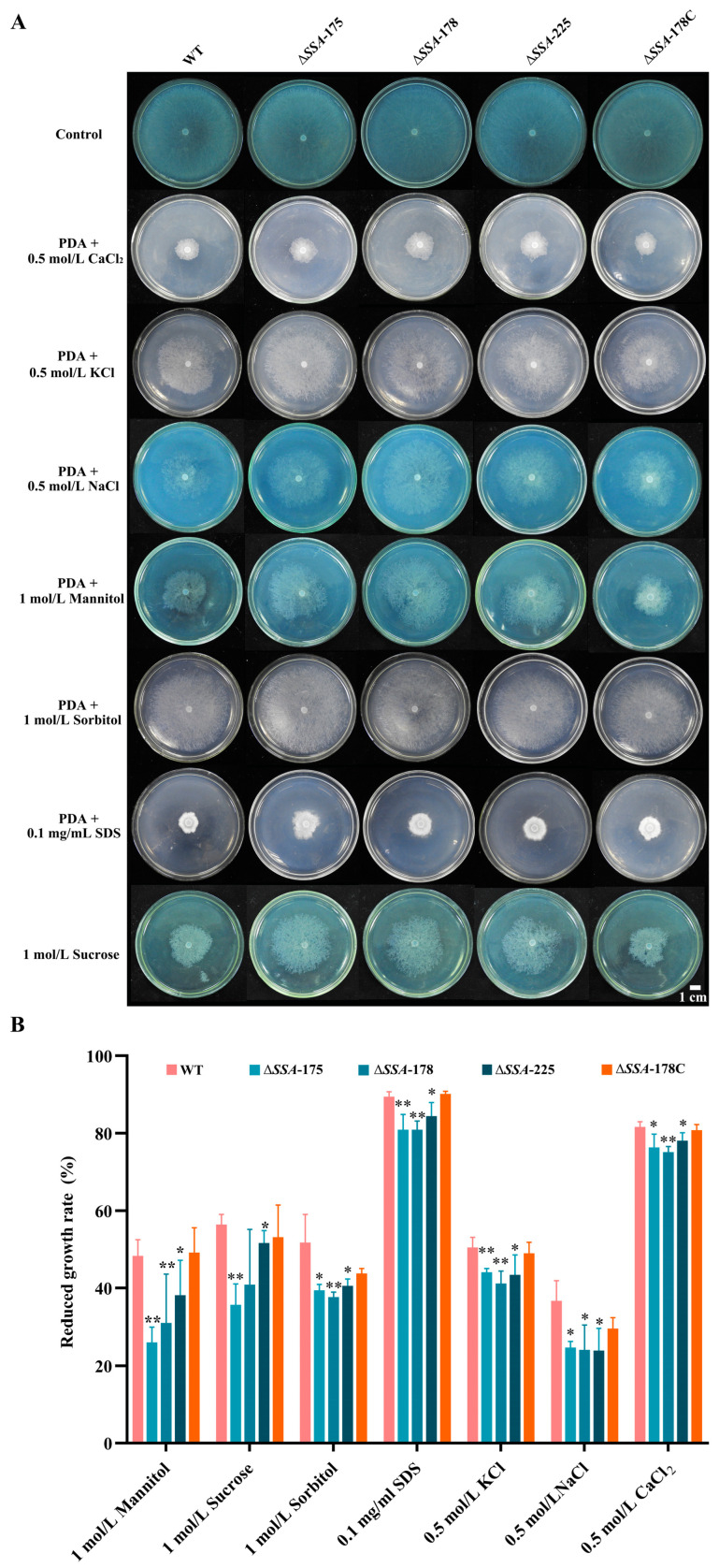
Response of the WT as well as the disruption and complementary mutants to chemical stresses. (**A**) Colonies of different strains on PDA amended with different chemicals (20 °C, 48 h); note the difference in colony size among the WT and the mutants. (**B**) Histogram showing the reduced growth rates of the WT and the mutants in treatments with different stress chemicals. * and ** represent significant differences between the WT and an investigated mutant at *p* < 0.05 and *p* < 0.01, respectively, according to Student’s *t* test.

The WT, the disruption mutants Δ*SSA*-175, Δ*SSA*-178, and Δ*SSA*-225, and the complementary mutant Δ*SSA*-178C grew rapidly on PDA alone at rates ranging from 22.1 to 22.3 mm/d. However, in the presence of the stress chemicals, the growth rates of the WT and the mutants were reduced to 2.2–17.0 mm/d (Figure 5A). The results also showed that the stress chemicals had different effects on the WT, the complementary mutant, and the disruption mutants regarding the extent of growth rate reduction. On PDA amended with NaCl, sorbitol, mannitol, KCl, sucrose, CaCl_2_ and SDS, the average growth rates were reduced by 33%, 48%, 49%, 50%, 55%, 81%, and 90% (compared to their growth rates on PDA alone), respectively, for WT and Δ*SSA*-178C, whereas the growth rates were reduced by 24%, 39%, 32%, 43%, 43%, 77% and 82%, respectively, for Δ*SSA*-175, Δ*SSA*-178 and Δ*SSA*-225 (Figure 5B). Statistical analysis showed that in response to each stress chemical, the disruption mutants had significantly lower (*p* < 0.05 or 0.01) percentages of growth rate reduction than those for WT and Δ*SSA*-178C (Figure 5B), indicating that the *SSA* disruption mutants were less sensitive than the WT and the complementary mutant to the investigated chemical stresses.

### 3.5. Effect of SSA Disruption on the Response of S. sclerotiorum to H_2_O_2_ Stresses

**Figure 6:**
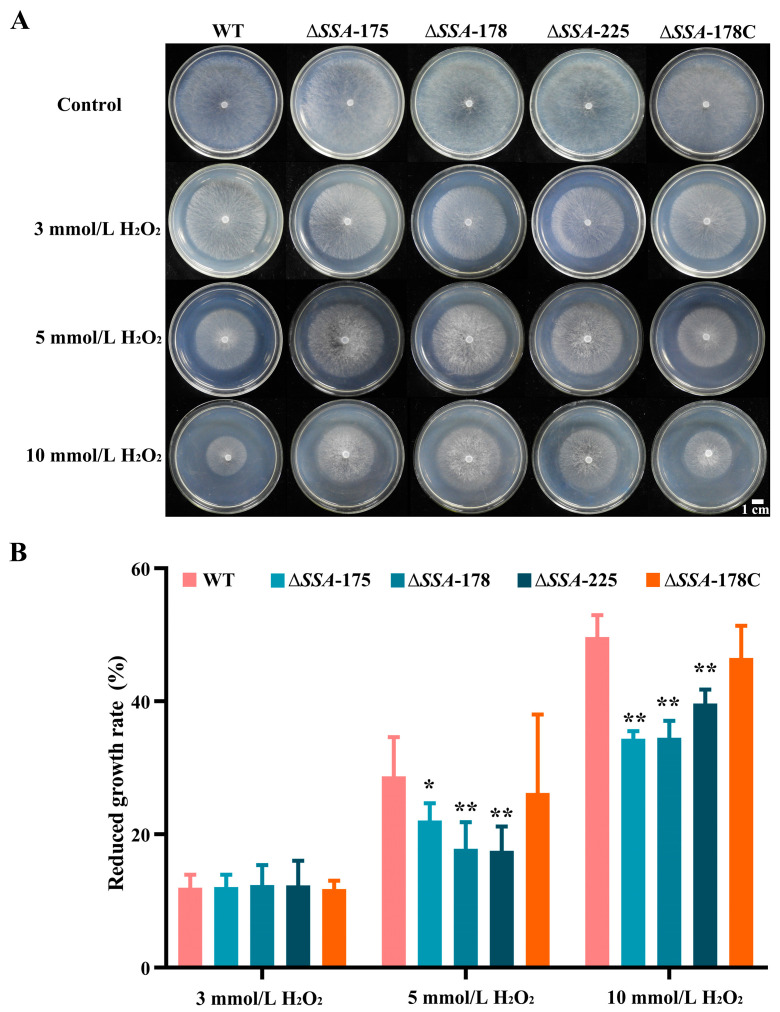
Response of the WT as well as the disruption and complementary mutants to the H_2_O_2_ stress. (**A**) Colonies of different strains on PDA amended with different concentrations of H_2_O_2_ (20 °C, 48 h); note the difference in colony size among the WT and the mutants. (**B**) Histogram showing the reduced growth rates of the WT and the mutants in the treatments with different concentrations of H_2_O_2_ stresses. * and ** represent significant differences between the WT and an investigated mutant at *p* < 0.05 and *p* < 0.01, respectively, according to Student’s *t* test.

The WT, Δ*SSA*-175, Δ*SSA*-178, Δ*SSA*-225, and Δ*SSA*-178C grew rapidly on PDA alone at rates of about 22 mm/d; they colonized the entire dish after incubation for 48 h (Figure 6A). In the presence of H_2_O_2_ (3, 5, 10 mmol/L), however, growth of these strains was inhibited to 11.2 to 19.6 mm/d; as a result, the dishes were partially colonized by these strains (Figure 6A). In cultures with H_2_O_2_ at 3 mmol/L, the growth rates of these five strains were reduced by ~12% without significant difference (*p* > 0.05) between each mutant and WT in the average percentage of growth rate reduction (Figure 6B). In cultures with H_2_O_2_ at 5 and 10 mmol/L, the growth rates of WT and Δ*SSA*-178C reduced by 27% and 48%, respectively; the values were higher than those (19% and 36%) for the three disruption mutants, respectively (Figure 6B). Statistical analysis indicated that under each concentration of H_2_O_2_, Δ*SSA*-178C did not significantly (*p* > 0.05) differ from WT in the value of growth rate reduction; however, the disruption mutants significantly differed from the WT in the value of growth rate reduction, indicating that the *SSA* disruption mutants were more resistant to H_2_O_2_ than the WT and the complementary mutant.

### 3.6. Effect of SSA Disruption on the Pathogenicity of S. sclerotiorum

**Figure 7:**
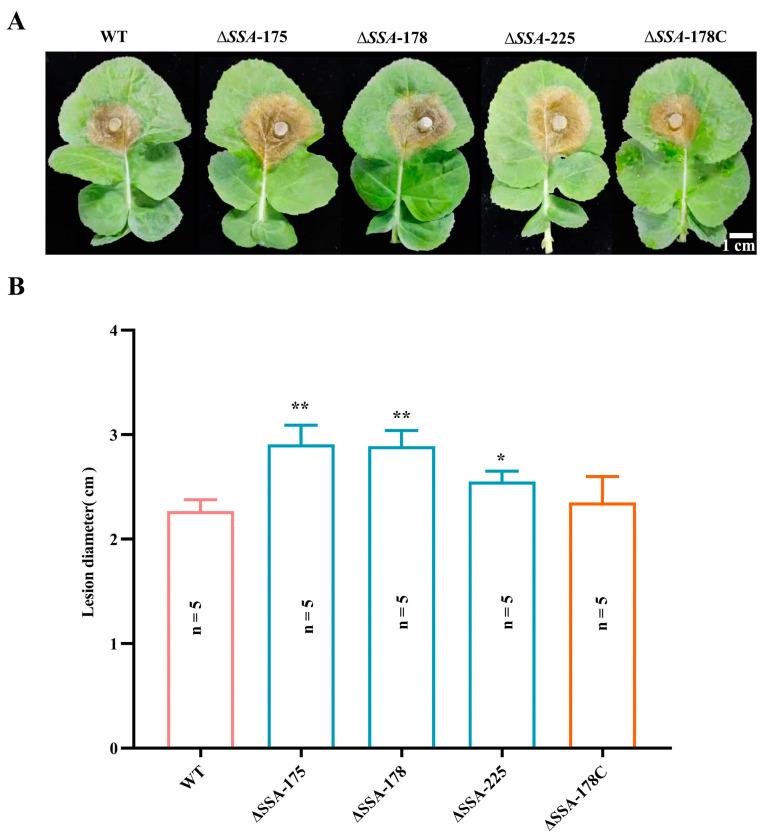
Pathogenicity of the WT as well as the disruption and complementary mutants on leaves of oilseed rape. (**A**) Five leaves showing the necrotic lesions caused by the WT and the mutants (20 °C, 48 h). (**B**) Histogram showing the average leaf lesion diameters. * and ** represent significant differences between the WT and an investigated mutant at *p* < 0.05 and *p* < 0.01, respectively, according to Student’s *t* test.

In humid conditions (20 °C, 48 h), WT, Δ*SSA*-175, Δ*SSA*-178, Δ*SSA*-225, and Δ*SSA*-178C infected leaves of oilseed rape and formed necrotic lesions around the inoculation plugs (Figure 7A). The WT and the complementary mutant formed lesions with average diameters of 22.2 and 22.9 mm, respectively; these measurements were significantly (*p* < 0.05 or 0.01) smaller than those of the lesions formed by the disruption mutants, which had average diameters ranging from 26.2 to 30.5 mm (Figure 7B). This comparison suggests that *SSA* negatively regulates pathogenicity of *S. sclerotiorum*.

### 3.7. Effect of SSA Disruption on the Resistance of S. sclerotiorum to Mycoparasitism by C. minitans

**Figure 8:**
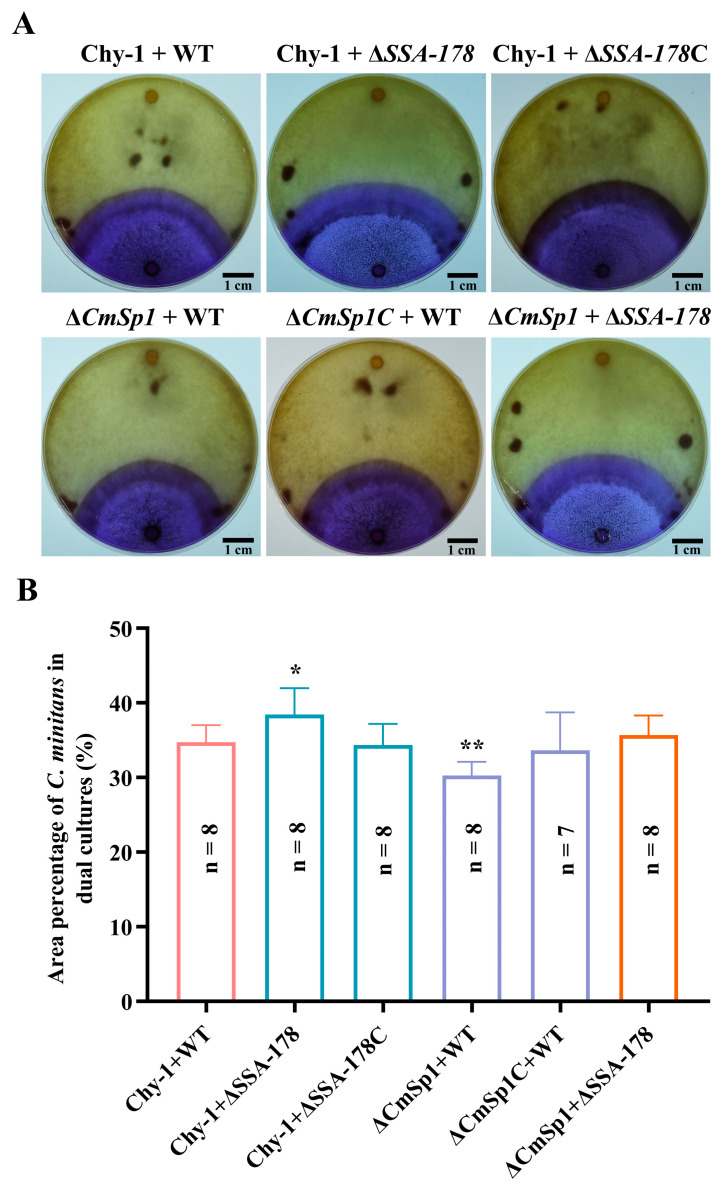
The aggressiveness of *C. minitans* in mycoparasitic invasion of the colonies of *S. sclerotiorum* in the dual cultures. (**A**) Six dual cultures on PDA amended with bromophenol blue (20 °C, 12 d); note the yellow color area colonized by *S. sclerotiorum* due to production of oxalic acid and the blue color area colonized by *C. minitans* due to degradation of oxalic acid. (**B**) Histogram showing the percentages of the *C. minitans*-colonized area. * and ** represent significant differences between the WT and an investigated mutant at *p* < 0.05 and *p* < 0.01, respectively, according to Student’s *t* test.

On PDA amended with the pH indicator bromophenol blue, dual cultures of *S. sclerotiorum* and *C. minitans* showed two contrasting colors (e.g., yellow and blue) which indicate colonization by *S. sclerotiorum* (e.g., production of oxalic acid) and *C. minitans* (e.g., degradation of oxalic acid), respectively, and the change of the color from yellow to blue in the dual cultures reflects the invasion of the *S. sclerotiorum* colonies by *C. minitans* [33]. The size of the blue area (BA) in the dual cultures varied not only with *C. minitans* WT Chy-1, Δ*CmSp1,* and Δ*CmSp1C*, but also with WT, Δ*SSA*-178, and Δ*SSA*-178C (Figure 8A). The six types of dual cultures had the average BA size which accounted for 30% to 38% of the total size of the Petri dishes (Figure 8B); the dual culture Chy-1 + Δ*SSA*-178 had the largest BA size (38%), whereas the dual culture Δ*CmSp1* + WT had the smallest BA size (30%). This comparison suggests that the WT of *C. minitans* Chy-1 is more aggressive than Δ*CmSp1* in invasion of the colonies of WT; the colonies of the disruption mutant Δ*SSA*-178 of *S. sclerotiorum* are less resistant than those of WT and Δ*SSA*-178C to invasion by *C. minitans.* Therefore, *SSA* positively affects the resistance of the colonies of *S. sclerotiorum* to mycoparasitism by *C. minitans.*

## 4. Discussion

This study revealed that SSA in *S. sclerotiorum* 1980 is an agglutinin with 100% identity to SSA in *S. sclerotiorum* S1954 [21]. *SSA* in *S. sclerotiorum* was successfully disrupted, alongside three disruption mutants (Δ*SSA*-175, Δ*SSA*-178, Δ*SSA*-225). Moreover, one of the disruption mutants, namely Δ*SSA*-178, was complemented with the *SSA* gene, and the complementary mutant Δ*SSA*-178C was then generated. These mutants as well as the WT are essential research materials for evaluating the biological functions of *SSA*.

Agglutinins are glycoproteins; they are synthesized early in fungal growth and exported outside, accumulating on the hyphal cell wall where they are activated to perform certain biological functions [17]. Agglutinins can specifically recognize carbohydrates, thereby playing a cell-to-cell adhesion role, thus modulating cell differentiation, cell recognition, and interaction [16,38,39]. In the present study, we found that disruption of *SSA* did not affect the mycelial growth of *S. sclerotiorum* on PDA; however, it affected sclerotial formation (through development) on PDA (through delayed sclerotial maturity) and on autoclaved carrot slices (it reduced sclerotial number, but increased sclerotial size and weight). It is well recognized that fungal sclerotia are a texturally hard, multicellular and nutrient-rich structure that can survive for a long time in adverse environments. Sclerotial development is a complicated process usually consisting of at least three stages, namely initiation, development, and maturation [40]. At the sclerotial initiation and development stages, fungal hyphal cells usually aggregate, and cell-to-cell recognition may become involved to form sclerotial primordia. This study observed that *SSA* disruption mutants were delayed for sclerotial maturity in the PDA cultures (Figure 3A), and formed less but larger sclerotia than the WT and the complementary mutant in the carrot cultures (Figure 4). These results suggest that SSA may become involved in sclerotial formation. On the other hand, the *SSA* disruption mutants were not completely blocked (suppressed) during sclerotial formation, implying that besides SSA, other agglutinins or signalling pathways may be involved in the sclerotial formation of *S. sclerotiorum*. We used the amino acid sequence of SSA to search other homologs in the genome of *S. sclerotiorum* 1980, and two homologs, namely XP001584968.1 and XP001587104.1, were identified (Figure S2). This result implies that physical contact signaling may participate in the sclerotial development or maturation in *S. sclerotiorum*.

Previous studies have shown that *S. sclerotiorum* is a typical necrotrophic plant pathogen [41] as it owns multiple plant-attacking weapons such as cell wall-degrading enzymes (CWDEs) and oxalic acid (OA) [42]. This study found that the WT, *SSA* disruption mutants, and the complementary mutant caused necrotic lesions on leaves of oilseed rape (Figure 7A). This result suggests that disruption of *SSA* did not completely eliminate the pathogenicity of *S. sclerotiorum*. The disruption mutants might retain the capability to produce CWDEs, as indicated by maceration of leaf tissues (Figure 7A), and to produce OA, as shown by the presence of the yellow color in PDA cultures amended with bromophenol blue (Figure 8A). However, compared to the WT and the complementary mutant, the *SSA* disruption mutants produced significantly larger (*p* < 0.05 or 0.01) leaf lesions on the leaves of oilseed rape. This result suggests that the disruption of *SSA* can enhance the aggressiveness of *S. sclerotiorum* in infecting leaves of oilseed rape. There are two possible reasons for this result: one is enhanced production of the pathogenesis-related chemical elements such as CWDEs, and the other one is reduced sensitivity to chemical, osmotic, and oxidative stresses from the leaves, as the disruption mutants became less sensitive to NaCl, KCl, CaCl_2_, sorbitol, mannitol, sucrose, SDS, and H_2_O_2_.

*C. minitans* is an obligate mycoparasite of *S. sclerotiorum*; it can attack both hyphae and sclerotia, resulting in sclerotial collapse and hyphal lysis, respectively [43,44]. Previous studies have shown that the mechanisms involved in mycoparasitism include production of extracellular enzymes, including chitinase, glucanases, and proteases [29,45], and elimination of OA toxicity via the degradation of OA [33]. Dual cultural assays have shown that invasion of the colonies of *S. sclerotiorum* by *C. minitans* causes the succession of *S. sclerotiorum* with *C. minitans* through the mycoparasitic interaction of *C. minitans* on *S. sclerotiorum* [46]. This study observed that *C. minitans* invaded the colonies of *S. sclerotiorum* with and without *SSA* in the dual cultures of the two fungi (Figure 8A). This result suggests that SSA is probably not the key agglutinin for determining the mycoparasitic specificity of *C. minitans*. Interestingly, the colonies of the *SSA* disruption mutants showed more susceptibility than those of the WT and the *SSA* complementary mutant to the *C. minitans* invasion, implying that SSA may positively modulate resistance to the mycoparasitism of *C. minitans*.

In summary, this study obtained three *SSA* disruption mutants (Δ*SSA*-175, Δ*SSA*-178, Δ*SSA*-225) and the complementary mutant Δ*SSA*-178C. On PDA, the disruption mutants did not differ from WT and Δ*SSA*-178C in their growth rate, but they did affect sclerotial development. On autoclaved carrot slices, they formed fewer but larger sclerotia than WT and Δ*SSA*-178C. The disruption mutants became less sensitive to seven chemical stresses and H_2_O_2_ stresses; however, they became more aggressive in infecting leaves of oilseed rape, and more susceptible to mycoparasitism by *C. minitans*, compared to WT and Δ*SSA*-178C. Therefore, *SSA* positively regulates sclerotial development and resistance to mycoparasitism by *C. minitans*, and negatively regulates pathogenicity and responses to abiotic environmental stresses.

## Data Availability

Not applicable.

## Supplementary Materials

The following supporting information can be downloaded at: https://www.mdpi.com/article/10.3390/jof9070737/s1, Figure S1: Plasmid map of pGNW; Figure S2: Phylogenetic analysis of *SSA*; Table S1: Primers used in this study.

## References

[1] Liang X.F., Rollins J.A. (2018). Mechanisms of broad host range necrotrophic pathogenesis in *Sclerotinia sclerotiorum*. Phytopathology.

[2] Xia S.T., Xu Y., Hoy R., Zhang J.L., Qin L., Li X. (2020). The notorious soilborne pathogenic fungus *Sclerotinia sclerotiorum*: An update on genes studied with mutant analysis. Pathogens.

[3] Harper G.E., Frampton C.M., Stewart A. (2002). Factors influencing survival of sclerotia of *Sclerotium cepivorum* in New Zealand soils. N. Z. J. Crop. Hortic. Sci..

[4] Cubeta M.A., Cody B.R., Kohli Y., Kohn L.M. (1997). Clonality of *Sclerotinia sclerotiorum* on infected cabbage in eastern North Carolina. Phytopathology.

[5] Attanayake R.N., Xu L.S., Chen W.D. (2019). *Sclerotinia sclerotiorum* populations: Clonal or recombining?. Trop. Plant Pathol..

[6] Amselem J., Cuomo C.A., van Kan J.A.L., Viaud M., Benito E.P., Couloux A., Coutinho P.M., de Vries R.P., Dyer P.S., Fillinger S. (2011). Genomic analysis of the necrotrophic fungal pathogens *Sclerotinia sclerotiorum* and *Botrytis cinerea*. PLoS Genet..

[7] Derbyshire M.C., Denton-Giles M. (2016). The control of sclerotinia stem rot on oilseed rape (*Brassica napus*): Current practices and future opportunities. Plant Pathol..

[8] Yoshimi A., Miyazawa K., Abe K. (2016). Cell wall structure and biogenesis in Aspergillus species. Biosci. Biotechnol. Biochem..

[9] Rodicio R., Heinisch J.J. (2010). Together we are strong—Cell wall integrity sensors in yeasts. Yeast.

[10] Levin D.E. (2011). Regulation of cell wall biogenesis in *Saccharomyces cerevisiae*: The cell wall integrity signaling pathway. Genetics.

[11] Jung U.S., Levin D.E. (1999). Genome-wide analysis of gene expression regulated by the yeast cell wall integrity signalling pathway. Mol. Microbiol..

[12] Jung U.S., Sobering A.K., Romeo M.J., Levin D.E. (2002). Regulation of the yeast Rlm1 transcription factor by the Mpk1 cell wall integrity MAP kinase. Mol. Microbiol..

[13] Singh R.S., Tiwary A.K., Kennedy J.F. (1999). Lectins: Sources, activities, and applications. Crit. Rev. Biotechnol..

[14] Singh R.S., Walia A.K. (2014). Microbial lectins and their prospective mitogenic potential. Crit. Rev. Microbiol..

[15] Singh R.S., Bhari R., Kaur H.P. (2011). Characteristics of yeast lectins and their role in cell-cell interactions. Biotechnol. Adv..

[16] Varrot A., Basheer S.M., Imberty A. (2013). Fungal lectins: Structure, function and potential applications. Curr. Opin. Struct. Biol..

[17] Stratford M., Carter A.T. (1993). Yeast flocculation: Lectin synthesis and activation. Yeast.

[18] Al-Mahmood S., Giummely P., Bonaly R., Delmotte F., Monsigny M. (1988). *Kluyveromyces bulgaricus* yeast lectins. Isolation of N-acetylglucosamine and galactose-specific lectins: Their relation with flocculation. J. Biol. Chem..

[19] Kellens J.T.C., Goldstein I.J., Peumans W.J. (1992). Lectins in different members of the Sclerotiniaceae. Mycol. Res..

[20] Candy L., Van Damme E.J.M., Peumans W.J., Menu-Bouaouiche L., Erard M., Rouge P. (2003). Structural and functional characterization of the GalNAc/Gal-specific lectin from the phytopathogenic ascomycete *Sclerotinia sclerotiorum* (Lib.) de Bary. Biochem. Biophys. Res. Commun..

[21] Van Damme E.J.M., Nakamura-Tsuruta S., Hirabayashi J., Rouge P., Peumans W.J. (2007). The *Sclerotinia sclerotiorum* agglutinin represents a novel family of fungal lectins remotely related to the *Clostridium botulinum* non-toxin haemagglutinin HA33/A. Glycoconj. J..

[22] Sulzenbacher G., Roig-Zamboni V., Peumans W.J., Rouge P., Van Damme E.J.M., Bourne Y. (2010). Crystal structure of the GalNAc/Gal-specific agglutinin from the phytopathogenic ascomycete *Sclerotinia sclerotiorum* reveals novel adaptation of a beta-trefoil domain. J. Mol. Biol..

[23] Alborzi Z., Zibaee A., Sendi J.J., Ramzi S. (2015). Effect of *Sclerotinia sclerotiorum* agglutinin on digestive α-amylase of *Pieris brassicae* L. (Lepidoptera: Pieridae). Rom. J. Biochem..

[24] Hamshou M., Smagghe G., Shahidi-Noghabi S., De Geyter E., Lannoo N., van Damme E.J.M. (2010). Insecticidal properties of *Sclerotinia sclerotiorum* agglutinin and its interaction with insect tissues and cells. Insect Biochem. Mol. Biol..

[25] Shen Y., De Schutter K., Walski T., Van Damme E.J.M., Smagghe G. (2017). Toxicity, membrane binding and uptake of the *Sclerotinia sclerotiorum* agglutinin (SSA) in different insect cell lines. Vitr. Cell. Dev. Biol..

[26] Van Toor R.F., Jaspers M.V., Stewart A. (2005). Effect of soil microorganisms on viability of sclerotia of *Ciborinia camelliae*, the causal agent of camellia flower blight. N. Z. J. Crop. Hortic. Sci..

[27] Brown A.J.P., Brown G.D., Netea M.G., Gow N.A.R. (2014). Metabolism impacts upon *Candida* immunogenicity and pathogenicity at multiple levels. Trends Microbiol..

[28] Gow N.A.R., Latge J.P., Munro C.A. (2017). The fungal cell wall: Structure, biosynthesis, and function. Microbiol. Spectr..

[29] Ren L., Li G.Q., Han Y.C., Jiang D.H., Huang H.C. (2007). Degradation of oxalic acid by *Coniothyrium minitans* and its effects on production and activity of β-1, 3-glucanase of this mycoparasite. Biol. Control.

[30] Hu Y.M., Yang L., Li G.Q. (2009). Optimization of culture conditions for production of chitinase by the mycoparasite *Coniothyrium minitans*. Chin. J. Biol. Control.

[31] Xie X.L., Yang L., Wu M.D., Zhang J., Li G.Q. (2016). Culture condition and characterization of factors affecting activity of the extracellular proteases produced by mycoparasite *Coniothyrium minitans*. Chin. J. Biol. Control.

[32] Wang Y.C., Yu H., Xu Y.P., Wu M.D., Zhang J., Tsuda K., Liu S.L., Jiang D.H., Chen W.D., Wei Y.D. (2023). Expression of a Mycoparasite Protease in Plant Petals Suppresses the Petal-Mediated Infection by Necrotrophic Pathogens. http://ssrn.com/abstract=4423202.

[33] Zeng L.M., Zhang J., Han Y.C., Yang L., Wu M.D., Jiang D.H., Chen W.D., Li G.Q. (2014). Degradation of oxalic acid by the mycoparasite *Coniothyrium minitans* plays an important role in interacting with *Sclerotinia sclerotiorum*. Environ. Microbiol..

[34] Yu H. (2016). Cloning and Functional Analysis of Extracellular Serine Protease Gene in the Mycoparasite Coniothyrium minitans.

[35] Möller E.M., Bahnweg G., Sandermann H., Geige H.H. (1992). A simple and efficient protocol for isolation of high molecular weight DNA from filamentous fungi, fruit bodies, and infected plant tissues. Nucleic Acids Res..

[36] Xie C., Shang Q.N., Mo C.M., Xiao Y.N., Wang G.F., Xie J.T., Jiang D.H., Xiao X.Q. (2022). Early secretory pathway-associated proteins SsEmp24 and SsErv25 are involved in morphogenesis and pathogenicity in a filamentous phytopathogenic fungus. mBio.

[37] Zhou Y.J., Song J.J., Wang Y.C., Yang L., Wu M.D., Li G.Q., Zhang J. (2022). Biological characterization of the melanin biosynthesis gene *Bcscd1* in the plant pathogenic fungus *Botrytis cinerea*. Fungal Genet. Biol..

[38] Manning J.C., Romero A., Habermann F.A., Caballero G.G., Kaltner H., Gabius H.J. (2017). Lectins: A primer for histochemists and cell biologists. Histochem. Cell Biol..

[39] Willaert R.G. (2018). Adhesins of yeasts: Protein structure and interactions. J. Fungi.

[40] Erental A., Dickman M.B., Yarden O. (2008). Sclerotial development in *Sclerotinia sclerotiorum*: Awakening molecular analysis of a “Dormant” structure. Fungal Biol. Rev..

[41] Bolton M.D., Thomma B.P.H.J., Nelson B.D. (2006). *Sclerotinia sclerotiorum* (Lib.) de Bary: Biology and molecular traits of a cosmopolitan pathogen. Mol. Plant Pathol..

[42] Marciano P., di Lenna P., Margo P. (1983). Oxalic acid, cell-wall degrading enzymes and pH in pathogenesis and their significance in the virulence of two *Sclerotinia sclerotiorum* isolates on sunflower. Physiol. Plant Pathol..

[43] Huang H.C., Kokko E.G. (1987). Ultrastructure of hyperparasitism of *Coniothyrium minitans* on sclerotia of *Sclerotinia sclerotiorum*. Can. J. Bot..

[44] Huang H.C., Kokko E.G. (1988). Penetration of hyphae of *Sclerotinia sclerotiorum* by *Coniothyrium minitans* without the formation of appressoria. J. Phytopathol..

[45] Giczey G., Kerényi Z., Fülöp L., Hornok L. (2001). Expression of *cmg1*, an exo-β-1,3-glucanase gene from *Coniothyrium minitans*, increases during sclerotial parasitism. Appl. Environ. Microbiol..

[46] Huang Y.B., Xie X.L., Yang L., Zhang J., Li G.Q., Jiang D.H. (2011). Susceptibility of *Sclerotinia sclerotiorum* strains different in oxalate production to infection by the mycoparasite *Coniothyrium minitans*. World J. Microbiol. Biotechnol..

