# Supplementary material for: Sclerotinia sclerotiorum Agglutinin Modulates Sclerotial Development, Pathogenicity and Response to Abiotic and Biotic Stresses in Different Manners
Source: J Fungi (Basel). 2023 Jul 10;9(7):737. doi: 10.3390/jof9070737 (PMC10381867; doi:10.3390/jof9070737)
Supplement: Supplementary file 1 [file jof-09-00737-s001.zip › jof-2471575-supplementary.pdf]

Journal of
*Fungi*

MDPI

*Supplementary Material*

# *Sclerotinia sclerotiorum* Agglutinin Modulates Sclerotial Development, Pathogenicity and Response to Abiotic and Biotic Stresses in Different Manners

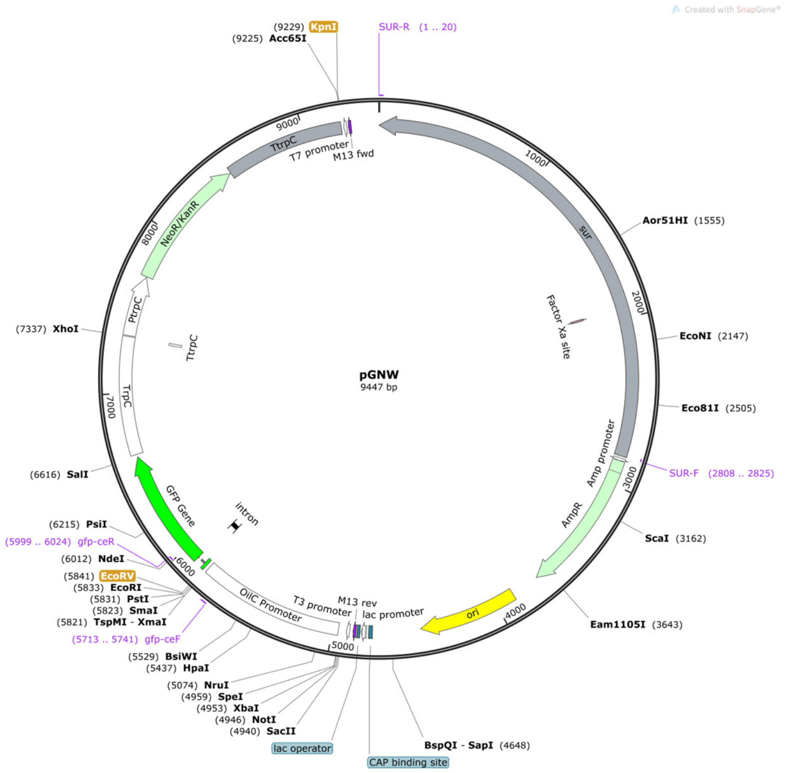


**Figure S1.** Plasmid map of pGNW.

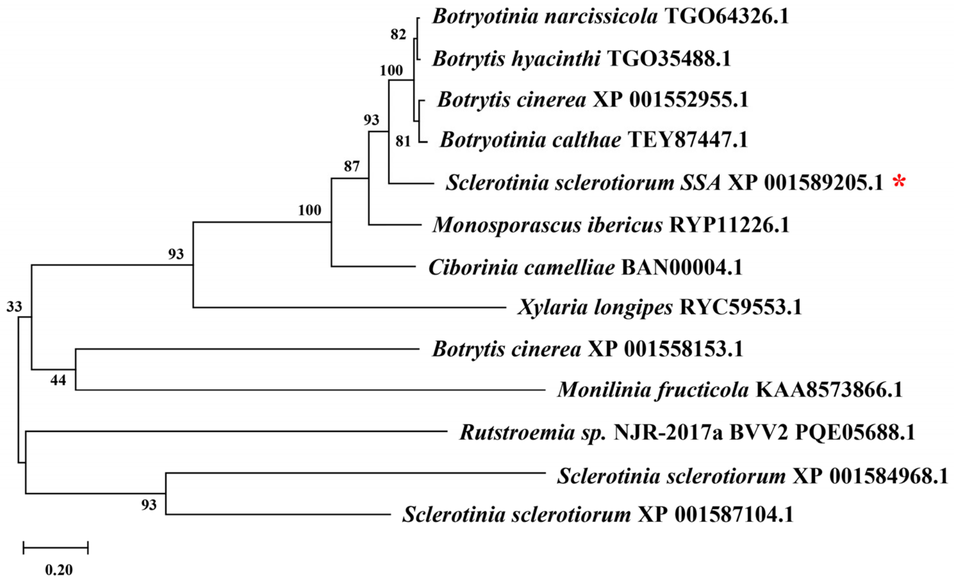


**Figure S2.** Phylogenetic analysis of *SSA*.

**Table S1.** Primers used in this study.

| Primer | Primer Sequence (5′-3′) |
|---|---|
| *SSA*-HyF | GTAAAACGACGGCCAGTGCCAAGCTTTGCAGTGCTAATCTT-GGGCA |
| *SSA*-HyR | ATTGAGTGATGACTTGTGGAGTCGACCTCCTTCAA-TATCATCTTCT |
| *SSA*-ygF | ATCCTTCTTTCTAGAGGATCCCCGGGATGGTTGCG A ATGC CGAT |
| *SSA*-ygR | TCCGTAAAGTTGGGGAATGAGAGCTCATGACCATGATTAC-GAATTC |
| gR-F | CTTAGCCAGACGAGCGGG |
| Hy-R | GACGAGTGCTGGGGCG |
| *SSA*-UpF | CACAATCGATCCAAGGATCCAGTTGGGATTTCCAC-TGTCCTG |
| *SSA*-UpR | ACCTCACCCTTGGAAACCATCTTGGCGTCAAAGTACCACTT |
| *SSA*-DownF | TTTAGAGGTAATCCTTCTTTATGGTTGCGAATGCCGAT |
| *SSA*-DownF | ACTCACTATAGGGCGAATT-GTAATGTCTAAGTCCCGTGATCCG |
| NeoF | GGGAAGGGACTGGCTGCTATTG |
| NeoR | AAAAGCGGCCATTTTCCACCAT |
| *SSA*F | ATGGGCTTTAAGGGCGTTGGT |
| *SSA*R | CTACTTGGCGTCAAAGTACCAC |
| HYG-F | ATGAAAAAGCCTGAACTCACCGC |
| HYG-R | CTATTCCTTTGCCCTCGGAC |
| NeO-F | CGATACCGTAAAGCACGAGGAAG |
| NeO-R | CGGCTATGACTGGGCACAACA |
| *SSA*f | ATGGGCTTTAAGGGCGTTGGT |
| *SSA*r | CTACTTGGCGTCAAAGTACCAC |
| HYGf | GATGTTGGCGACCTCGTATT |
| HYGr | TCGTTATGTTTATCGGCACTTT |
| NeOf | ATGATTGAACAAGATGGATTGCAC |
| NeOr | AAAAGCGGCCATTTTCCACCAT |
| P*SSA*F | CGGACCAAAAACAATAACGA |
| P*SSA*R | GATAGGTGCCCTCACATAGC |
